# Travel to low- and middle-income countries and travellers’ diarrhoea increase risk of mismatching antimicrobial therapy for urinary tract infection

**DOI:** 10.1093/jtm/taaf025

**Published:** 2025-03-28

**Authors:** Anu Patjas, Anu Kantele

**Affiliations:** Meilahti Vaccine Research Center, MeVac, Department of Infectious Diseases, University of Helsinki and Helsinki University Hospital, FI-00029 HUS Helsinki, Finland; Human Microbiome Research Unit, University of Helsinki, Haartmaninkatu 3, FI-00290 Helsinki, Finland; Finnish Multidisciplinary Center of Excellence in Antimicrobial Resistance Research, FIMAR, University of Helsinki, Haartmaninkatu 3, FI-00290 Helsinki, Finland; Meilahti Vaccine Research Center, MeVac, Department of Infectious Diseases, University of Helsinki and Helsinki University Hospital, FI-00029 HUS Helsinki, Finland; Human Microbiome Research Unit, University of Helsinki, Haartmaninkatu 3, FI-00290 Helsinki, Finland; Finnish Multidisciplinary Center of Excellence in Antimicrobial Resistance Research, FIMAR, University of Helsinki, Haartmaninkatu 3, FI-00290 Helsinki, Finland

**Keywords:** Antimicrobial resistance, urinary tract infection, ESBL-PE, *Escherichia coli*, trimethoprim, LMIC travel, Enterobacterales

## Abstract

**Background:**

Travel to low- and middle-income countries (LMICs) increases the risk of urinary tract infections (UTIs), including those caused by extended-spectrum beta-lactamase-producing Enterobacterales (ESBL-PE). Focusing on international travel, we explored resistance profiles of urinary ESBL-PE and non-ESBL-PE isolates in a low antimicrobial resistance prevalence country and factors associated with UTI treatment failure.

**Methods:**

During 2015–19, we recruited 18–65-year-old individuals with recent ESBL-PE UTI and a respective cohort of those with non-ESBL-PE UTI to complete questionnaires on symptoms, antibiotic therapies and treatment failure risk factors. We compared uropathogens’ resistance profiles amongst patients with or without LMIC travel history and conducted multivariable analyses to identify factors contributing to mismatching antimicrobial treatment (uropathogen resistant to the initial antimicrobial used) and clinical failure.

**Results:**

Amongst non-ESBL-PE UTI patients (n = 187), trimethoprim resistance was more common in isolates from individuals with recent LMIC travel (8/19, 42.1%) compared to those without (30/167, 18.0%) [odds ratio (OR) 3.3, compatibility interval (CI) 95% 1.2–9.0]. ESBL-PE isolates (n = 130) showed no differences in resistance profiles with respect to LMIC travel history.

In the group non-ESBL-PE UTI, risk factors included microbiological mismatching recent LMIC travel [adjusted odds ratio (AOR) 3.6, CI 95% 1.0–12.7] and travellers’ diarrhoea (AOR 7.1, CI 95% 1.1–45.6); no factors were significantly associated with mismatching in the group ESBL-PE UTI. As risk factors for clinical failure, in the group non-ESBL-PE UTI, we identified microbiological mismatching (AOR 15.2, CI 95% 4.0–57.9), and renal/bladder disease (AOR 5.2, CI 95% 1.1–23.2), and in the group ESBL-PE UTI, microbiological mismatching (AOR 8.1, CI 95% 2.6–24.7).

**Conclusions:**

LMIC travel increases the risk of nonmatching empiric antimicrobials, concurring with increased trimethoprim resistance rates amongst the non-ESBL-PE isolates. Our data suggest that UTI patients with recent LMIC travel should not be empirically treated with trimethoprim and, when possible, urinary culturing is warranted.

## Introduction

In 2019, antimicrobial resistance (AMR) was estimated to cause up to 1.27 million AMR-attributable and 4.9 million AMR-associated deaths worldwide.^1^ The burden is greatest in low- and middle-income countries (LMICs) but spreads from there with international travel and trade across the globe. In the World Health Organization (WHO) Europe region alone, an annual estimate of 133 000 AMR-attributable and 541 000 AMR-associated deaths was recently reported.^2^  *Escherichia coli*, the most common pathogen in urinary tract infections (UTIs),^3^ caused more deaths than any other pathogen.^1^ Consistent with the globally increasing AMR, extended-spectrum beta-lactamase-producing Enterobacterales (ESBL-PE) and other multidrug-resistant (MDR) bacteria have become increasingly prevalent amongst clinical uropathogens.^4^

International travel has been indicated as a risk factor for both UTI^5^^,^^6^ and having ESBL-PE as a cause of UTI,^6–9^ according with the numerous investigations showing high rates of intestinal ESBL-PE acquisition by travellers to LMICs.^10–14^ Our recent study reported that 41% of travel-acquired colonizing ESBL-*E. coli* strains have virulence factors defining them as uropathogenic *E. coli* (UPEC).^15^ Furthermore, travel-acquired ESBL-PEs, including ESBL-UPECs are often co-resistant to many non-beta-lactam antimicrobials.^15–17^

The international guidelines advise that empiric antimicrobial therapy for UTIs should follow local resistance patterns.^18^^,^^19^ For cystitis, trimethoprim/co-trimoxazole, nitrofurantoin, pivmecillinam or fosfomycin are recommended as the first-line agents, and for pyelonephritis fluoroquinolones, co-trimoxazole or extended-spectrum beta-lactams.^18^^,^^19^ However, as the urinary pathogens in many LMICs exhibit higher resistance rates to many antimicrobials compared to low-prevalence countries like Finland,^4^ our local guidelines—aligned with the international recommendations—may not be suitable for travellers returning from LMICs who may have contracted their uropathogen abroad. Currently, no treatment guidelines specifically address travellers contracting UTI during or shortly after visiting high-AMR prevalence areas.

We recently reported UTI treatment failure rates amongst Finnish non-ESBL-PE and ESBL-PE UTI patients.^20^ To obtain data for empiric choices of UTI antimicrobials for those returning from high AMR prevalence areas, we set out to explore i) resistance profiles of ESBL and non-ESBL urinary pathogens in Finland by LMIC travel history; ii) risk factors for mismatching initial antimicrobial UTI treatment (uropathogen resistant to the initial antimicrobial used) and iii) risk factors for UTI treatment failure.

## Methods

### Participants and study design

**Figure 1:**
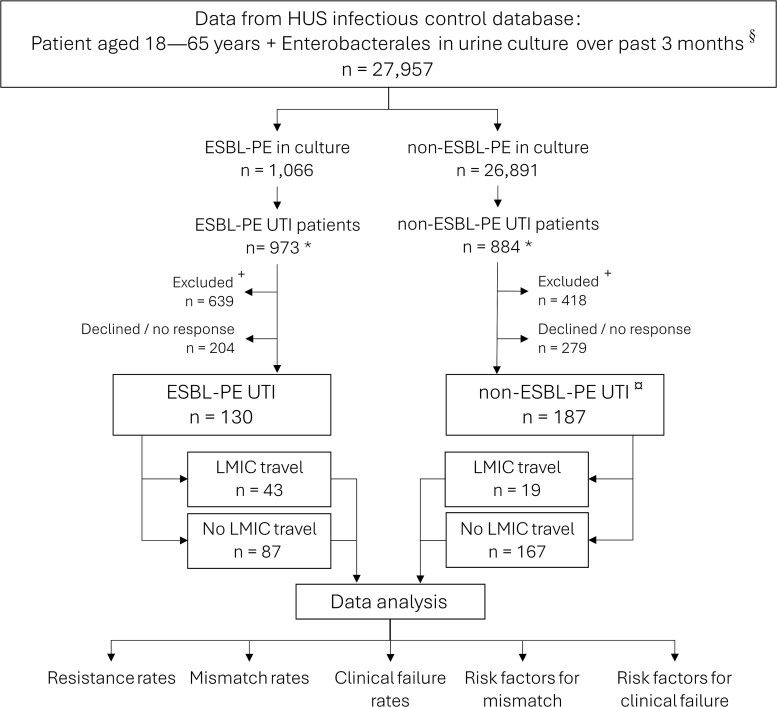
Study protocol comparing community-acquired Enterobacterales-UTI patients with and without LMIC travel over the past year, with separate analyses for the groups ESBL-PE UTI and non-ESBL-PE UTI. The analyses examined uropathogen resistance profiles, rates of mismatching antimicrobial treatment, and clinical failure rates, as well as potential risk factors for mismatching antimicrobial (uropathogen resistant to initial antimicrobial used) and clinical failure of UTI treatment. ^§^Data from 9 retrievals. ^*^Deceased patients and multiple samples per patient removed; compared to ESBL-PE UTI patients, we selected a respective number of non-ESBL-PE UTI patients. ^+^The following exclusion criteria were applied: i) previous ESBL-PE colonization (n = 263), ii) residency in a nursing home (n = 42), iii) hospitalization for ˃24 h over past month (n = 36), iv) permanent residency abroad over past year (n = 8), v) inability to participate (e.g. language barrier, aphasia or dementia) (n = 162), vi) lack of contact information in HUS database or not reached (n = 519) and vii) asymptomatic bacteriuria unless subject was pregnant (n = 27). ^
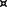
^Data on LMIC travel history missing from one participant. Abbreviations: HUS, HUS Helsinki University Hospital; ESBL-PE, extended-spectrum beta-lactamase -producing Enterobacterales; UTI; urinary tract infection; LMIC, low- and middle-income countries.

We retrieved data on UTI patients aged 18–65 years from the HUS Helsinki University Hospital (hereafter HUS) infection control database over a 3-month period at a time, between 1 December 2015 and 31 May 2019. Data collection occurred nine times throughout the recruitment period. Those with suspected nosocomial UTI or previous ESBL-PE colonization were excluded. For each subset, we recruited all patients with a community-onset ESBL-PE UTI and a corresponding number of those with non-ESBL-PE UTI to fill in questionnaires on UTI symptoms and treatment, the questions covering potential risk factors for treatment failure. Data on antimicrobial regimens and blood culture results were retrieved from HUS patient records for those giving separate consent. All patients were initially contacted by phone. Questionnaires were completed electronically, on paper, or through a phone interview, as the participant preferred. Participant eligibility was evaluated at each step of the recruitment process. All patients provided written informed consent. Figure 1 illustrates the study design. HUS Ethical Committee approved the study protocol (408/13/03/00/2015).

### Definitions

All definitions in the present study followed those of the previous studies on the same patient cohort.^6^^,^^20^

#### Urinary tract infections and recurrence

UTI was defined as at least 10^5^ CFU/mL bacterial growth in the urine sample and symptoms compatible with cystitis or pyelonephritis.^19^^,^^21^ Afebrile (<38°C) patients with negative or no blood cultures and symptoms compatible with cystitis (frequency, dysuria, urgency) were categorized as cystitis; febrile (≥38°C) UTIs and those with bacteraemia were defined as pyelonephritis. We also included pregnant women with asymptomatic bacteriuria, as this condition warrants antimicrobial treatment.^19^

Recurrent UTI was defined according to international guidelines: at least two UTI episodes over 6 months or three over a year.^22^

#### Uropathogen identification and antimicrobial susceptibility

Uropathogen identification and antimicrobial susceptibility were determined in routine clinical practices at the HUS laboratory (HUSLAB) following the European Committee on Antimicrobial Susceptibility Testing (EUCAST) criteria^23^; the detailed process has been described elsewhere.^20^ For the present study, intermediate antimicrobial susceptibility (I) was categorized as susceptible (S).

#### Microbiological mismatch/match

The treatment was defined as mismatching if the uropathogen was resistant and matching if the uropathogen was susceptible or of intermediate susceptibility (I) *in vitro* to the first antimicrobial the patient received. Those with data missing on the first antimicrobial regimen but having a uropathogen susceptible to all antimicrobials in the primary testing panel (cephalexin, cefuroxime, trimethoprim, mecillinam, nitrofurantoin and ciprofloxacin) were defined as matches. Others with no data on the first antimicrobial regimen were excluded from the matching analyses.

As for ESBL-PE UTI patients with a pathogen intermediately susceptible (I) to pivmecillinam, cases receiving a standard dose of 200 mg three times a day were categorized as mismatches, and those receiving higher dose of 400 mg three times a day (recommended for ESBL-PE cystitis)^24^ as matches.

For those with two urinary pathogens in the index sample, the matching analyses followed the pathogen listed first in the laboratory report.

#### Clinical failure

Clinical failure was defined as a need to switch the initial antimicrobial treatment or receive additional antimicrobials for the index UTI episode or persisting symptoms (>6 weeks) reported in the questionnaire. A transition from intravenous to peroral antimicrobial as part of routine care was not considered a failure. In the clinical failure analyses, we excluded asymptomatic pregnant participants, those who did not receive any antimicrobial regimens, and those whose symptoms resolved spontaneously without antimicrobials.

#### Travellers’ diarrhoea

For travellers’ diarrhoea (TD), we used the WHO criteria for diarrhoea: passage of three or more loose or liquid stools per day, or more frequently than is usual for the individual.^25^

### Statistical analyses

SPSS Statistics Software (version 29.0.0.0, IBM Corp., USA) and Stata (version 18.0, StataCorp LLC, USA) were used for statistical analyses. For univariable models, *P*-values were calculated with Chi square, binary logistic and Firth logistic regression analyses. For risk factor analyses, variables with *P* < 0.2 in univariable analyses were selected for multivariable models by binary logistic regression. The final multivariable models were determined using backward selection, guided by the Akaike information criterion (AIC).

## Results

In total 159 592 positive urinary cultures were screened for Enterobacterales isolates and age of 18–65 years, resulting in 27 957 isolates, including 1066 ESBL-PE. For the detailed process, see Figure 1. The final study population comprised 130 ESBL-PE UTI patients and 187 non-ESBL-PE UTI patients—a roughly corresponding number chosen to achieve comparable group sizes. The two cohorts were analyzed separately, as detailed below.

### Demographics and uropathogens

Of all participants, 89.3% (283/317) were women; the median age was 51 years. Upper UTIs were recorded for 21.8% (69/317), and recurrent UTIs for 31.1% (98/317). Travel to LMICs within the past year was reported by 10.2% (19/186) of patients in the group non-ESBL-PE UTI and 33.1% (43/130) in the group ESBL-PE UTI, for a total of 19.6% (62/316).

*E. coli* was the identified pathogen in 94.0% (298/317) and *Klebsiella pneumoniae* in 6.0% (19/317) of patients. A co-infection was recorded for 2.0% (6/298) and 10.5% (2/19) of the infections, respectively.

For more detailed data on demographics and uropathogens with respect to LMIC travel history, see Table 1.

**Table 1:** Demographics of UTI patients with and without a history of LMIC travel over the past year. Data are provided separately for those with non-ESBL-PE and ESBL-PE UTI over the past 3 months

	non-ESBL-PE UTI				ESBL-PE UTI			
	All (%)^a^ n = 187	No LMIC travel (%) n = 167	LMIC travel (%) n = 19	*P*-value	All (%) n = 130	No LMIC travel (%) n = 87	LMIC travel (%) n = 43	*P*-value
Sex (md = 0)				0.755				0.122
women	174 (93.0)	155 (92.8)	18 (94.7)		109 (83.8)	76 (87.4)	33 (76.7)	
men	13 (7.0)	12 (7.2)	1 (5.3)		21 (16.2)	11 (12.6)	10 (23.3)	
Age, median (IQR) (md = 0)	51 (35–60)	50 (35–60)	56 (42–64)	0.496	49 (34–57)	46 (34–57)	51 (34–60)	0.446
Underlying disease or condition (md = 0)								
cardiovascular or respiratory disease	66 (35.3)	61 (36.5)	5 (26.3)	0.378	43 (33.1)	28 (32.2)	15 (34.9)	0.758
gastrointestinal disease	9 (4.8)	9 (5.4)	0 (0.0)	0.300	6 (4.6)	5 (5.7)	1 (2.3)	0.382
diabetes mellitus	21 (11.2)	20 (12.0)	1 (5.3)	0.381	9 (6.9)	5 (5.7)	4 (9.3)	0.452
renal insufficiency or transplant	4 (2.1)	3 (1.8)	1 (5.3)	0.324	1 (0.8)	1 (1.1)	0 (0.0)	0.480
bladder dysfunction or any kind of catheter	13 (7.0)	10 (6.0)	3 (15.8)	0.115	5 (3.8)	5 (5.7)	0 (0.0)	0.109
immunosuppression	12 (6.4)	11 (6.6)	1 (5.3)	0.824	10 (7.7)	8 (9.2)	2 (4.7)	0.360
Recurrent UTIs (md = 2)	63 (34.1)	56 (33.9)	6 (31.6)	0.837	35 (26.9)	26 (29.9)	9 (20.9)	0.279
Pathogen (md = 0)				0.445				0.202
* E. coli* ^b^	176 (94.1)	157 (94.0)	17 (89.5)		122 (93.8)	80 (92.0)	42 (97.7)	
* Klebsiella pneumoniae ^c^*	11 (5.9)	10 (6.0)	2 (10.5)		8 (6.2)	7 (8.0)	1 (2.3)	
Upper UTI (md = 0)	30 (16.0)	28 (16.8)	2 (10.5)	0.483	39 (30.0)	21 (24.1)	18 (41.9)	0.038

Abbreviations: ESBL-PE, extended-spectrum beta-lactamase-producing Enterobacterales; UTI, urinary tract infection; LMIC, low- and middle-income countries; md, missing data; IQR, interquartile range. *P*-values calculated by Chi square test.

^a^Data on LMIC travel history missing from 1 participant; numbers and percentages reported for all non-ESBL-PE UTI patients.

^b^Two co-infections with *Enterococcus faecalis*, one with *Citrobacter* species, one with *Staphylococcus saprophyticus* and one with *Streptococcus agalactiae.*

^c^One co-infection with *Enterococcus faecalis.*

### Antimicrobial susceptibility by isolates

The resistance rates in the group non-ESBL-PE were generally low (<10%), apart from trimethoprim with resistance rate of 20.4% (38/186), the rates significantly higher amongst those with a history of LMIC travel than those without (Table 2). As for ESBL-PE, low resistance rates (<10%) were only found for mecillinam and nitrofurantoin.

**Table 2:** AMR amongst urinary isolates of UTI patients with versus without a travel history to LMICs over the past year

	non-ESBL-PE UTI					ESBL-PE UTI				
	All (%) (n = 187)	LMIC travel (%) (n = 19)	no LMIC travel (%) (n = 167)	OR (CI 95%)^a^	*P*-value^a^	All (%) (n = 130)	LMIC travel (%) (n = 43)	no LMIC travel (%) (n = 87)	OR (CI 95%)^a^	*P*-value^a^
Mecillinam (md = 1)	2 (1.1)	0 (0.0)	2 (1.2)	1.7 (0.1–36.7)	0.736	7 (5.4)	2 (4.8)	5 (5.7)	0.8 (0.2–4.4)	0.817
Trimethoprim^b^ (md = 0)	38 (20.3)	8 (42.1)	30 (18.0)	3.3 (1.2–9.0)	0.018	86 (66.2)	29 (67.4)	57 (65.5)	1.1 (0.5–2.4)	0.827
Nitrofurantoin (md = 5)	3 (1.6)	0 (0.0)	3 (1.8)	1.2 (0.1–23.6)	0.916	5 (3.9)	0 (0.0)	5 (5.7)	0.2 (0.01–3.3)	0.244
Fluoroquinolones (md = 0)	12 (6.4)	1 (5.3)	11 (6.6)	0.8 (0.1–6.5)	0.824	61 (46.9)	21 (48.8)	40 (46.0)	1.1 (0.5–2.3)	0.759
Cephalexin (md = 20)	1 (0.6)	0 (0.0)	1 (0.7)	2.7 (0.1–67.7)	0.554	130 (100.0)	43 (100.0)	87 (100.0)	N/A	N/A
Resistant to all antimicrobials in primary panel^c^ (md = 20)	0 (0.0)	0 (0.0)	0 (0.0)	N/A	N/A	1 (0.8)	0 (0.0)	1 (0.8)	0.7 (0.03–16.6)	0.802

Abbreviations: ESBL-PE, extended-spectrum beta-lactamase -producing *Enterobacterales*; UTI, urinary tract infection; LMIC, low- and middle-income countries; OR, odds ratio; CI, compatibility interval; md, missing data; N/A, not applicable.

^a^ *P*-values calculated by Chi square test. For variables with quasi-complete separation, Firth logistic regression was used for calculation of *P*-value, OR and CI.

^b^Resistance to co-trimoxazole is not reported due to many isolates not tested for it.

^c^Cephalexin, cefuroxime, trimethoprim, mecillinam, nitrofurantoin and ciprofloxacin.

### Antimicrobial susceptibility by LMIC travel history

In the group non-ESBL-PE UTI, trimethoprim resistance was more frequent amongst isolates from individuals with a history of LMIC travel (42.1%, 8/19) compared to those who had only travelled to high-income areas or had no travel history (18.0%, 30/167) [odds ratio (OR) 3.3, compatibility interval (CI) 95% 1.2–9.0]. For other antimicrobials, the differences by LMIC travel history were not significant (Table 2). In the group ESBL-PE UTI, no differences were recorded in resistance patterns between those with and without recent LMIC travel (Table 2). We found no significant differences in antimicrobial susceptibility between those with and without TD over the past year in either group (data not shown).

### Microbiological mismatching rates with respect to LMIC travel history

In the group non-ESBL-PE UTI, the mismatch rate amongst those with a history of LMIC travel was 21.1% (4/19), and amongst those without it, 6.9% (11/159) (OR 3.6, CI 95% 1.0–12.7, *P* = 0.036). In the group ESBL-PE UTI, the respective figures were 48.0% (12/25) and 55.4% (31/56) (OR 0.7, CI 95% 0.3–1.9, *P* = 0.540).

### Clinical failure rates with respect to LMIC travel history

In the group non-ESBL-PE UTI, the clinical failure rate was 29.4% (5/17) amongst those with a history of LMIC travel and 16.2% (25/154) (OR 2.2, CI 95% 0.7–6.6, *P* = 0.175) amongst those without. In the group ESBL-PE UTI, the respective figures were 41.0% (16/39) and 35.0% (28/80) (OR 1.3, CI 95% 0.6–2.8, *P* = 0.523).

### Risk factor analysis 1: Factors associated with microbiological mismatching

In the group non-ESBL-PE UTI, on the basis of univariable analyses, age, LMIC travel over the past year and TD over the past year were selected to the multivariable models. All participants with TD had a history of LMIC travel: thus, these two variables did not fit into the same model, and two separate final models were created (Table 3). In these two analyses, either LMIC over the past year (*P* = 0.047), or TD over the past year (*P* = 0.038) were identified as independent risk factors for mismatching therapy.

**Table 3:** Risk factor analysis 1. Potential risk factors for microbiological mismatching amongst non-ESBL-PE UTI patients: uni- and multivariable analyses. Two separate multivariable models were used: first one including LMIC travel but not TD, the second one including TD but not LMIC travel due to the multicollinearity of these two variables

Risk factor	Total (%) (n = 179)	Microbiological mismatch (%) (n = 15)	Microbiological match (%) (n = 164)	Univariable analyses		Multivariable model 1		Multivariable model 2	
				OR (CI 95%)	*P*-value	AOR (CI 95%)	*P*-value	AOR (CI 95%)	*P*-value
Women (md = 0)	167 (93.3)	13 (7.8)	154 (92.2)	0.4 (0.1–2.1)	0.297				
Age, years (median (IQR)), 10 years increase (md = 0)	51 (35–60)	56 (43–61)	49.5 (35–60)	1.0 (1.0–1.1)	0.175	Eliminated^a^		1.0 (1.0–1.1)	0.144
*Klebsiella pneumoniae* in urine culture (vs *E. coli*) (md = 0)	12 (6.7)	1 (8.3)	11 (91.7)	1.0 (0.1–8.3)	0.995				
Bloodstream infection (md = 62)	4 (3.4)	0 (0.0)	4 (100.0)	1.1 (0.1–21.8)^b^	0.512^b^				
Upper UTI (md = 0)	30 (16.8)	1 (3.3)	29 (96.7)	0.3 (0.04–2.6)	0.297				
Immunosuppression (md = 0)	12 (6.7)	2 (16.7)	10 (83.3)	2.4 (0.5–12.0)	0.297				
Renal or bladder disease/dysfunction (md = 1)	16 (9.0)	2 (12.5)	14 (87.5)	1.6 (0.3–8.0)	0.542				
Diabetes (md = 0)	20 (11.1)	2 (10.0)	18 (90.0)	1.2 (0.3–6.0)	0.782				
Recurrent UTIs (md = 2)	60 (33.9)	5 (8.3)	55 (91.7)	1.0 (0.3–3.0)	0.961				
Antibiotic for non-UTI indication past year (md = 7)	55 (32.0)	4 (7.3)	51 (92.7)	0.8 (0.3–2.8)	0.776				
Hospitalization past year^c^ (md = 1)	36 (20.2)	2 (5.6)	34 (94.4)	0.6 (0.1–2.7)	0.492				
LMIC travel past year (md = 1)	19 (10.7)	4 (21.1)	15 (78.9)	3.6 (1.0–12.7)	0.047	3.6 (1.0–12.7)	0.047		
TD past year (md = 0)	6 (3.4)	2 (33.3)	4 (66.7)	6.2 (1.0–36.8)	0.047			7.1 (1.1–45.6)	0.038
LMIC travel by household members past year (md = 2)	4 (2.3)	1 (25.0)	3 (75.0)	3.8 (0.4–38.8)	0.262				
Pets (md = 2)	56 (31.6)	5 (8.9)	51 (91.1)	1.1 (0.4–3.3)	0.883				
Weekly fish meals (md = 0)	111 (62.0)	7 (6.3)	104 (93.7)	0.5 (0.2–1.5)	0.208				

Abbreviations: OR, odds ratio; CI, compatibility interval; AOR; adjusted odds ratio; md, missing data; UTI, urinary tract infection; LMIC, low- and middle-income countries; TD, travellers’ diarrhoea.

Mismatching analyses were based on the resistance profile of the pathogen listed first in the laboratory report. Amongst the five non-ESBL-PE co-infections included in the analyses, three were considered clinically less significant (*Enterococcus faecalis* as the pathogen listed second with growth <10^5^ CFU/mL); one had similar resistance profiles for both pathogens listed first and second (*Citrobacter* species 10^5^ CFU/mL); one UTI had *E. coli* (susceptible to all antimicrobials in the panel) as the pathogen listed first and *K. pneumoniae* (resistant to trimethoprim, susceptible to others; >10^5^ CFU/mL) as the pathogen listed second; data on the initial antimicrobial agent was missing, and the treatment was categorized as matching based on the pathogen listed second; one co-infection was excluded from the matching analyses due to missing antimicrobial data.

Univariable *P*-values calculated by binary logistic regression analysis.

^a^Variable eliminated from multivariable model 1 by backward selection.

^b^Calculated by Firth logistic regression analysis.

^c^For ˃24 h.

In the group ESBL-PE UTI, none of the variables reached statistical significance although a tendency was identified for *K. pneumoniae* isolates and diabetes (Supplementary Table S1).

### Risk factor analysis 2: Factors associated with clinical failure

In the group non-ESBL-PE UTI, we identified as factors associated with clinical failure two factors: microbiological mismatching (*P* < 0.001) and renal/bladder disease or dysfunction (*P* = 0.030) (Table 4). In addition, the following factors were included in the final multivariable model but failed to reach statistical significance: higher age, antimicrobial regimens for non-UTI indication over the past year, *K. pneumoniae* isolate, and co-infection (Table 4).

**Table 4:** Risk factor analysis 2. Potential risk factors for clinical failure amongst non-ESBL-PE UTI patients: uni- and multivariable analyses

Risk factor	Total (%) (n = 172)	Clinical failure (%) (n = 30)	Clinical cure (%) (n = 142)	Univariable analyses		Multivariable analysis	
				OR (CI 95%)	*P*-value	AOR (CI 95%)	*P*-value
Women (md = 0)	159 (92.4)	26 (16.4)	133 (83.6)	0.4 (0.1–1.5)	0.198	Eliminated^a^	
Age, years [median (IQR)], 10 years increase (md = 0)	51 (35–51)	58 (47–64)	49 (35–60)	1.0 (1.0–1.0)	0.027	1.0 (1.0–1.1)	0.054
*Klebsiella pneumoniae* in urine culture (vs *E. coli*) (md = 0)	11 (6.4)	5 (45.5)	6 (54.5)	4.5 (1.3–16.0)	0.019	3.7 (0.8–17.4)	0.096
Co-infection (md = 0)	5 (2.9)	3 (60.0)	2 (40.0)	7.2 (1.3–38.1)	0.021	5.7 (0.7–47.3)	0.108
Bloodstream infection (md = 60)	4 (3.6)	1 (25.0)	3 (75.0)	1.5 (0.1–14.8)	0.746		
Upper UTI (md = 0)	29 (16.9)	6 (20.7)	23 (79.3)	1.3 (0.5–3.5)	0.614		
Immunosuppression (md = 0)	10 (5.8)	2 (20.0)	8 (80.0)	1.2 (0.2–5.9)	0.826		
Renal or bladder disease/dysfunction (md = 1)	13 (7.6)	6 (46.2)	7 (53.8)	4.6 (1.5–15.5)	0.009	5.2 (1.1–23.2)	0.030
Diabetes (md = 0)	18 (10.5)	5 (27.8)	13 (72.2)	2.0 (0.6–6.0)	0.229		
Recurrent UTIs (md = 1)	59 (34.5)	13 (22.0)	46 (78.0)	1.6 (0.7–3.5)	0.265		
Antibiotic for non-UTI indication past year (md = 7)	54 (32.7)	13 (24.1)	41 (75.9)	1.9 (0.8–4.3)	0.130	2.4 (0.9–6.3)	0.084
Hospitalization past year^b^ (md = 1)	36 (21.1)	4 (11.1)	32 (88.9)	0.5 (0.2–1.6)	0.260		
LMIC travel past year (md = 1)	17 (9.9)	5 (29.4)	12 (70.6)	2.2 (0.7–6.6)	0.183	Eliminated^a^	
TD past year (md = 0)	6 (3.5)	3 (50.0)	3 (50.0)	5.1 (1.0–26.9)	0.052	Eliminated^a^	
LMIC travel by household members past year (md = 2)	4 (0.2)	0 (0.0)	4 (100.0)	0.5 (0.03–9.5)^c^	0.642^c^		
Pets (md = 2)	52 (30.6)	10 (19.2)	42 (80.8)	1.2 (0.5–2.7)	0.719		
Weekly fish meals (md = 0)	110 (64.0)	18 (16.4)	92 (83.6)	0.8 (0.4–1.8)	0.620		
Microbiological mismatching (md = 5)	15 (9.0)	10 (66.7)	5 (33.3)	13.2 (4.1–42.6)	<0.001	15.2 (4.0–57.9)	<0.001

Abbreviations: OR, odds ratio; CI, compatibility interval; AOR; adjusted odds ratio; md, missing data; UTI, urinary tract infection; LMIC, low- and middle-income countries; TD, travellers’ diarrhoea.

Univariable *P*-values calculated by binary logistic regression analysis.

^a^Variables eliminated from final multivariable model by AIC.

^b^For ˃24 h.

^c^Calculated by Firth logistic regression analysis.

In the group ESBL-PE UTI, microbiological mismatching (*P* < 0.001) was identified as a risk factor; antimicrobials for non-UTI indication over the past year did not reach statistical significance (Supplementary Table S2).

## Discussion

As UTI is the most common extraintestinal infection caused by travel-acquired intestinal bacteria,^15^ we set out to explore in a low AMR-prevalence country the appropriateness and success of empirical treatment of UTI patients who had visited LMICs. These results add to our previous finding of an increased risk of ESBL-PE UTI amongst travellers to LMICs^6^: we now demonstrate higher rates of both microbiological mismatching and clinical failure amongst non-ESBL-PE UTI patients with recent travel to LMIC. Our data suggest that the current UTI guidelines should not be applied when treating returning LMIC travellers with UTI.

### Antimicrobial resistance and associations with LMIC travel

The resistance rates in this cohort were documented in our previous report.^20^ As expected,^15–17^ ESBL-PE isolates showed high resistance rates also to non-beta-lactam antibiotics, with particularly high rates for trimethoprim and fluoroquinolones; no differences were recorded by LMIC travel history within this group. However, as only 2.1–4.9% of UTIs in Finland are caused by ESBL-PE,^26^ co-resistance amongst ESBL-PE is of less consequence than that amongst non-ESBL-PE in our setting. Amongst non-ESBL-PE isolates, 20% were resistant to trimethoprim, i.e. the limit after which empiric treatment is no longer recommended^19^; the resistance levels for the other antimicrobials remained low. In comparisons concerning LMIC travels, only trimethoprim showed statistical significance with higher rates amongst those with LMIC travel (42%) than those without (18%) in the group non-ESBL-PE UTI, the data highlighting the role of LMIC travel in the acquisition of resistant uropathogens in low AMR-prevalence regions. Despite our lack of direct data on co-trimoxazole resistance—as it is not included in our hospitals’ primary testing panel—this finding probably applies to co-trimoxazole as well, since trimethoprim-resistant strains are often co-resistant to co-trimoxazole.^27^

Previous studies have demonstrated that both LMIC travel and TD are independent risk factors for colonization by intestinal ESBL-PE^10^^,^^11^^,^^13^^,^^14^ and fluoroquinolone-resistant Enterobacterales.^28^ More recently, both LMIC travel^6^^,^^7^ and TD^6^ have been described as factors associated with having ESBL-PE instead of non-ESBL-PE as a uropathogen. Less attention has been paid to LMIC travel and TD as risk factors for UTI caused by bacteria resistant to non-beta-lactams. A handful of studies have linked LMIC travel and LMIC origin to increased uropathogen resistance^9^^,^^29–33^ and risk of mismatching UTI treatment.^34^ None of those studies explored TD as a potential risk factor.

### Risk factor analysis 1: Factors associated with microbiological mismatching

Our study expands existing knowledge by identifying both LMIC travel and TD abroad as risk factors for microbiological mismatching amongst non-ESBL-PE UTI patients in a low AMR-prevalence country. These associations are most likely explained by the over twofold rate of trimethoprim resistance amongst non-ESBL-PE isolates from UTI patients with recent LMIC travel (18% vs 42%). Indeed, up to 90% resistance rates for antimicrobials recommended for UTIs—such as trimethoprim, co-trimoxazole and fluoroquinolones—have been reported in UPECs amongst locals in LMICs^4^^,^^35^; travellers to these regions have been shown to become colonized by such uropathogenic strains.^15^ Importantly, ESBL-PE strains—overrepresented after LMIC travel^10–14^—exhibit high rates of trimethoprim/co-trimoxazole and fluoroquinolone resistance.^15–17^ These findings indicate that urinary cultures should be obtained from all UTI patients potentially acquiring their uropathogens in high AMR-prevalence areas—locals and travellers alike. Furthermore, empirical treatment for LMIC travellers with UTI should prioritize antimicrobials other than trimethoprim/co-trimoxazole or fluoroquinolones.

Previous data from both LMICs and high-income countries consistently show low resistance rates to nitrofurantoin, fosfomycin and pivmecillinam amongst non-ESBL-PE and ESBL-PE strains.^16^^,^^35–39^ Likewise, nitrofurantoin resistance was rare amongst our isolates. Data on fosfomycin resistance were unavailable as this has not been routinely tested in Finland. Nitrofurantoin, fosfomycin and even higher dose pivmecillinam emerge as viable options for empirical UTI treatment in patients with a recent history of LMIC travel.

In our study population, mismatching therapy tended to be associated with older age in the group non-ESBL-PE UTI and with *Klebsiella* isolates in the group ESBL-PE UTI, though these associations did not reach statistical significance. Previous studies have reported an increased risk of mismatching antimicrobial therapy in elderly UTI patients,^30^^,^^40^ and higher resistance rates amongst *Klebsiella* species than *E. coli*.^1^^,^^4^ In our data, the lack of significance for these and other previously identified risk factors may reflect a particularly strong association of mismatched therapy with LMIC travel and/or TD in this setting.

### Risk factor analysis 2: Factors associated with clinical treatment failure

Factors associated with clinical failure fall into two categories: uropathogen-related factors (such as AMR) and host-related factors (either permanent or transient conditions),^41^^,^^42^ with many of these factors interrelated (e.g. prior antimicrobials and recurrent UTIs).

Amongst non-ESBL-PE UTI patients, we observed a higher rate of clinical failures in those with recent LMIC travel compared to those without. However, LMIC travel or TD—both identified as risk factors for microbiological mismatching—were eliminated from the final multivariable model by AIC. Instead, microbiological mismatching itself appeared to be the key predisposing factor for clinical failure in both groups non-ESBL-PE and ESBL-PE UTI. This suggests that the impact of LMIC travel on clinical failure is more likely mediated through resistant uropathogens rather than direct effects on host properties.

In the final multivariable model for non-ESBL-PE UTI, factors associated with clinical failure included co-infections, *K. pneumoniae* as uropathogen, higher age, urinary tract diseases and previous antimicrobial regimens (the latter also identified in the group ESBL-PE UTI): all these factors have been linked with UTI treatment failure also in previous investigations.^33^^,^^41–45^ However, none of the previous studies exploring risk factors for clinical failure of UTI have considered international travel as a potential risk factor.

### Limitations

Finnish and international guidelines do not advise urinary cultures for diagnosing uncomplicated cystitis.^19^^,^^21^ Our recruitment method relied on positive urinary cultures, which may have resulted in an overrepresentation of patients with upper, recurrent, or complicated UTIs, or pathogens with higher resistance profiles.

The mismatch rates might be slightly underestimated, since, for a small group of volunteers, antimicrobial therapy may have been initiated only after culture results were obtained, as discussed previously.^20^ However, as a rule, treatment is initiated without waiting for results. Conversely, clinical failure rates could be somewhat overestimated, as some changes in antimicrobial treatment may have been due to adverse effects rather than inefficacy. However, urinary antimicrobials are generally well-tolerated.^18^

The retrospective study design and reliance on questionnaire data introduce a potential recall bias. To mitigate this, we included only UTI patients with recent episodes (within the past three months), and with separate consent, supplemented the data on antimicrobial regimens using patient records.

## Conclusions

Our findings reveal an over twofold higher rate of trimethoprim resistance in non-ESBL-PE strains amongst patients with recent travel to LMIC than amongst those without such travel history. Furthermore, recent LMIC travel and TD emerged as independent risk factors for microbiological mismatching in non-ESBL-PE UTIs.

Based on these results, we recommend i) urine sampling from all cystitis patients with a recent LMIC travel due to increased risk of resistant uropathogens, and ii) given the high trimethoprim resistance rates in both travel-acquired ESBL-PE and non-ESBL-PE strains, avoiding empirical trimethoprim treatment in UTI patients with recent LMIC travel.

## Supplementary Material

Patjas_JTM_UTI_mismatch_travel_Supplements_070225_taaf025

## Data Availability

All relevant data are in the manuscript and its Supplementary material file. The individuals' data cannot be shared publicly due to research regulations. Further information directly from the authors.
