# Supplementary material for: Travel to low- and middle-income countries and travellers’ diarrhoea increase risk of mismatching antimicrobial therapy for urinary tract infection
Source: J Travel Med. 2025 Mar 28;32(4):taaf025. doi: 10.1093/jtm/taaf025 (PMC12118056; doi:10.1093/jtm/taaf025)
Supplement: Patjas_JTM_UTI_mismatch_travel_Supplements_070225_taaf025 [file patjas_jtm_uti_mismatch_travel_supplements_070225_taaf025.docx]

**Supplementary Table S1.** The results of the uni- and multivariable analyses on potential risk factors for microbiological mismatching among ESBL-PE UTI patients.

| Risk factor | Total (%)  n=81 | Microbiological mismatch (%)  n=43 | Microbiological match (%)  n=38 | Univariable analyses | | Multivariable analysis | |
| --- | --- | --- | --- | --- | --- | --- | --- |
|  |  |  |  | OR (CI 95%) | p value | AOR (CI 95%) | p value |
| Women (md=0) | 66 (81.5) | 34 (51.5) | 32 (48.5) | 0.7 (0.2–2.2) | 0.553 |  |  |
| Age, years (median (IQR)),  10 years increase (md=0) | 49 (34–57) | 50.5 (37–57) | 48 (33–56) | 1.0 (1.0–1.0) | 0.673 |  |  |
| *K. pneumoniae* in urine culture (vs *E. coli*) (md=0) | 6 (7.4) | 5 (83.3) | 1 (16.7) | 4.9 (0.6–43.7) | 0.157 | 7.8 (0.6–95.7) | 0.110 |
| Bloodstream infection (md=16) | 2 (3.1) | 2 (100.0) | 0 (0.0) | 4.3 (0.2–92.6)^a^ | 0.355^a^ |  |  |
| Upper UTI (md=0) | 28 (34.6) | 13 (46.4) | 15 (53.6) | 0.7 (0.3–1.7) | 0.384 |  |  |
| Immunosuppression (md=0) | 3 (3.7) | 2 (66.7) | 1 (33.3) | 1.8 (0.2–20.7) | 0.635 |  |  |
| Renal or bladder disease / dysfunction (md=0) | 4 (4.9) | 3 (75.0) | 1 (25.0) | 2.8 (0.3–27.9) | 0.386 |  |  |
| Diabetes (md=0) | 5 (6.2) | 1 (20.0) | 4 (80.0) | 0.2 (0.02–1.9) | 0.162 | 0.1 (0.01–1.6) | 0.111 |
| Recurrent UTIs (md=0) | 20 (24.7) | 11 (55.0) | 9 (45.0) | 1.1 (0.4–3.1) | 0.843 |  |  |
| Antibiotic for non-UTI indication past year (md=0) | 41 (50.6) | 21 (51.2) | 20 (48.8) | 0.9 (0.4–2.1) | 0.733 |  |  |
| Hospitalisation past year ^b^ (md=0) | 19 (23.5) | 8 (42.1) | 11 (57.9) | 0.6 (0.2–1.6) | 0.276 |  |  |
| LMIC travel past year (md=0) | 25 (30.9) | 12 (48.0) | 13 (52.0) | 0.7 (0.3–1.9) | 0.540 |  |  |
| TD past year (md=0) | 8 (9.9) | 3 (37.5) | 5 (62.5) | 0.5 (0.1–2.2) | 0.359 |  |  |
| LMIC travel by household members past year (md=0) | 5 (6.2) | 2 (40.0) | 3 (60.0) | 0.6 (0.1–3.6) | 0.569 |  |  |
| Pets (md=0) | 36 (44.4) | 20 (55.6) | 16 (44.4) | 1.2 (0.5–2.9) | 0.691 |  |  |
| Weekly fish meals (md=0) | 33 (40.7) | 16 (48.5) | 17 (51.5) | 0.7 (0.3–1.8) | 0.492 |  |  |

Abbreviations: OR, odds ratio; CI, compatibility interval; AOR; adjusted odds ratio; md, missing data; UTI, urinary tract infection; LMIC, low- and middle-income countries; TD, travellers’ diarrhoea

Mismatching analyses followed the resistance profile of the primary pathogen. None of the ESBL-PE co-infections were included in the matching analyses (missing antimicrobial data / no antimicrobial treatment).

Univariable p-values calculated by binary logistic regression analysis.

^a^ calculated by Firth logistic regression analysis

^b^ for more than 24 hours

**Supplementary Table S2.** The results of the uni- and multivariable analyses on potential risk factors for clinical failure among ESBL-PE UTI patients.

| Risk factor | Total (%)  n=119 | Clinical failure (%)  n=45 | Clinical cure (%)  n=74 | Univariable analyses | | Multivariable analysis | |
| --- | --- | --- | --- | --- | --- | --- | --- |
|  |  |  |  | OR (CI 95%) | p value | AOR (CI 95%) | p value |
| Women (md=0) | 100 (84.0) | 37 (37.0) | 63 (63.0) | 0.8 (0.3–2.2) | 0.674 |  |  |
| Age, years (median (IQR)),  10 years increase (md=0) | 49 (34–57) | 48 (33.5–56.5) | 48.5 (36–58) | 1.0 (1.0–1.1) | 0.574 |  |  |
| *K. pneumoniae* in urine culture (vs *E. coli*) (md=0) | 8 (6.7) | 5 (62.5) | 3 (37.5) | 3.0 (0.7–13.0) | 0.152 | Eliminated ^a^ |  |
| Co-infection (md=0) | 1 (0.8) | 1 (100.0) | 0 (0.0) | 5.0 (0.2–126.0)^b^ | 0.326^b^ |  |  |
| Bloodstream infection (md=43) | 3 (4.0) | 1 (33.3) | 2 (66.7) | 0.8 (0.1–9.8) | 0.898 |  |  |
| Upper UTI (md=0) | 37 (31.1) | 13 (35.1) | 24 (64.9) | 0.8 (0.4–1.9) | 0.686 |  |  |
| Immunosuppression (md=0) | 8 (6.7) | 3 (37.5) | 5 (62.5) | 1.0 (0.2–4.3) | 0.985 |  |  |
| Renal or bladder disease / dysfunction (md=0) | 5 (4.2) | 3 (60.0) | 2 (40.0) | 2.6 (0.4–16.0) | 0.312 |  |  |
| Diabetes (md=0) | 7 (5.9) | 2 (28.6) | 5 (71.4) | 0.6 (0.1–3.5) | 0.606 |  |  |
| Recurrent UTIs (md=0) | 33 (27.7) | 11 (33.3) | 22 (66.7) | 0.8 (0.3–1.8) | 0.533 |  |  |
| Antibiotic for non-UTI indication past year (md=3) | 60 (51.7) | 28 (46.7) | 32 (53.3) | 2.2 (1.0–4.7) | 0.046 | 2.5 (0.9–7.3) | 0.082 |
| Hospitalisation past year ^c^ (md=0) | 32 (26.9) | 14 (43.8) | 18 (56.3) | 1.4 (0.6–3.2) | 0.419 |  |  |
| LMIC travel past year (md=0) | 39 (32.8) | 16 (41.0) | 23 (59.0) | 1.2 (0.6–2.7) | 0.614 |  |  |
| TD past year (md=0) | 13 (10.9) | 7 (53.8) | 6 (46.2) | 2.1 (0.7–6.7) | 0.214 |  |  |
| LMIC travel by household members past year (md=0) | 13 (10.9) | 3 (23.1) | 10 (76.9) | 0.5 (0.1–1.8) | 0.255 |  |  |
| Pets (md=0) | 50 (42.0) | 20 (40.0) | 30 (60.0) | 1.2 (0.6–2.5) | 0.676 |  |  |
| Weekly fish meals (md=0) | 55 (46.2) | 20 (36.4) | 35 (63.6) | 0.9 (0.4–1.9) | 0.762 |  |  |
| Microbiological mismatching (md=40) | 43 (36.1) | 25 (58.1) | 18 (41.9) | 6.9 (2.4–20.2) | <0.001 | 8.1 (2.6–24.7) | <0.001 |

Abbreviations: OR, odds ratio; CI, compatibility interval; AOR; adjusted odds ratio; UTI, urinary tract infection; LMIC, low- and middle-income countries; TD, travellers’ diarrhoea

Univariable p-values calculated by binary logistic regression analysis.

^a^ variable eliminated from final multivariable model by AIC

^b^ calculated by Firth logistic regression analysis

^c^ for more than 24 hours
